# Supplementary material for: A large-scale genome-wide association meta-analysis for nevus count provides direct insights into the genetics of melanoma
Source: Nat Commun. 2026 Mar 10;17:3772. doi: 10.1038/s41467-026-70368-5 (PMC13106845; doi:10.1038/s41467-026-70368-5)
Supplement: Supplementary file 2 — Description of Additional Supplementary Files [file 41467_2026_70368_MOESM2_ESM.pdf]

Description of Additional Supplementary Files

File Name: Supplementary Data 1
Description: Summary statistics of pair-wise GWAS (GWAS-PW) analysis

File Name: Supplementary Data 2
Description: Summary statistics of prioritized 246 genes mapped based on positional eQTL and chromatin interaction mapping approaches

File Name: Supplementary Data 3
Description: Summary results of 29 nevus associated loci

File Name: Supplementary Data 4
Description: Summary statistics for significant genes (at threshold of multiple testing corrected $p < 2.64E-6$) identified in MAGMA gene based analysis

File Name: Supplementary Data 5.1
Description: Summary statistics for significant genes identified by TWAS using FUSION

File Name: Supplementary Data 5.2
Description: Summary statistics for significant genes identified by TWAS using SUMMIT

File Name: Supplementary Data 6
Description: Summary statistics for top 10 gene sets identified using MAGMA gene set analysis

File Name: Supplementary Data 7
Description: Summary of gene enrichment tests of 248 prioritized genes

File Name: Supplementary Data 8
Description: Z-test statistics for the statistical difference of sex-specific genome-wide significant loci in men and women
