# Supplementary material for: A large-scale genome-wide association meta-analysis for nevus count provides direct insights into the genetics of melanoma
Source: Nat Commun. 2026 Mar 10;17:3772. doi: 10.1038/s41467-026-70368-5 (PMC13106845; doi:10.1038/s41467-026-70368-5)
Supplement: Supplementary file 4 — Reporting Summary [file 41467_2026_70368_MOESM4_ESM.pdf]

nature portfolio

Corresponding author(s): G J M S R Jayasinghe
Matthe H Law

Last updated by author(s): Feb 13, 2026

Reporting Summary

Nature Portfolio wishes to improve the reproducibility of the work that we publish. This form provides structure for consistency and transparency in reporting. For further information on Nature Portfolio policies, see our Editorial Policies and the Editorial Policy Checklist.

Statistics

For all statistical analyses, confirm that the following items are present in the figure legend, table legend, main text, or Methods section.

n/a	Confirmed	
☐	☒	The exact sample size (*n*) for each experimental group/condition, given as a discrete number and unit of measurement
☐	☒	A statement on whether measurements were taken from distinct samples or whether the same sample was measured repeatedly
☐	☒	The statistical test(s) used AND whether they are one- or two-sided<br>*Only common tests should be described solely by name; describe more complex techniques in the Methods section.*
☐	☒	A description of all covariates tested
☐	☒	A description of any assumptions or corrections, such as tests of normality and adjustment for multiple comparisons
☐	☒	A full description of the statistical parameters including central tendency (e.g. means) or other basic estimates (e.g. regression coefficient) AND variation (e.g. standard deviation) or associated estimates of uncertainty (e.g. confidence intervals)
☐	☒	For null hypothesis testing, the test statistic (e.g. *F*, *t*, *r*) with confidence intervals, effect sizes, degrees of freedom and *P* value noted<br>*Give P values as exact values whenever suitable.*
☐	☒	For Bayesian analysis, information on the choice of priors and Markov chain Monte Carlo settings
☒	☐	For hierarchical and complex designs, identification of the appropriate level for tests and full reporting of outcomes
☒	☐	Estimates of effect sizes (e.g. Cohen's *d*, Pearson's *r*), indicating how they were calculated

Our web collection on statistics for biologists contains articles on many of the points above.

Software and code

Policy information about availability of computer code

Data collection	Data source for the GWAS analysis had been provided in the method section of the main text and supplementary notes.
Data analysis	The following analysis tools were used in our study: GCTA v.1.26, SAIGE v.1.1.4, POLMM: GRAB R package v.0.1.1 in R version 4.0.2, LDSC v1.0.0, METAL v.2020-05-05, DENTIST v.1.3.0.0, FUMA v.1.6.0, topr package in R version 4.3.1, TWAS FUSION, SUMMIT v.1.0.2, COLOC v.5.1.0.1 package in R v.4.2.0, GWAS-PW v.0.21, TwoSampleMR package in R version 4.3.1, SBayesRC, PLINK v.2.0

For manuscripts utilizing custom algorithms or software that are central to the research but not yet described in published literature, software must be made available to editors and reviewers. We strongly encourage code deposition in a community repository (e.g. GitHub). See the Nature Portfolio guidelines for submitting code & software for further information.

Data

Policy information about availability of data

All manuscripts must include a data availability statement. This statement should provide the following information, where applicable:

- Accession codes, unique identifiers, or web links for publicly available datasets
- A description of any restrictions on data availability
- For clinical datasets or third party data, please ensure that the statement adheres to our policy

Summary data for GWAS meta-analyses, including the sex-stratified meta-analyses, and nevus count PRS SNPs with weights, are available on Zenodo [https://doi.org/10.5281/zenodo.18537530]. All the individual GWAS summary statistics will be available from the corresponding authors upon request, subject to

regulatory approval from the relevant institution. Individual-level genotype and phenotype data used in the heritability analysis and PRS analysis are protected and not publicly available due to data privacy laws. The UK Biobank reference panel was accessed under application number 25331. Access to UK Biobank data can be obtained by applying to UK Biobank (https://www.ukbiobank.ac.uk/). The dataset of LD blocks used in GWAS-PW analysis is available from https://bitbucket.org/nygcresearch/ldetect-data. LD score reference data can be accessed through https://alkesgroup.broadinstitute.org/LDSCORE/eur_w_ld_chr.tar.bz2. FUMA provides the reference panels and datasets used in the described analysis; identifying loci, functional annotation, gene-based and gene-set analysis through https://fuma.ctglab.nl/. Pre-computed gene expression weights for GTEx skin tissues are available on the FUSION website (http://gusevlab.org/projects/fusion/). Pre-calculated gene expression models for eQTL whole blood data can be accessed through https://doi.org/10.17605/OSF.IO/7MXSA. SBayesRC provides LD data calculated from UK Biobank European ancestry and functional annotation data through https://github.com/zhilizheng/SBayesRC. GWAS for melanoma confirmed meta-analysis (with the exclusion of self-reported data from 23andMe and UK Biobank) is publicly available at dbGaP (phs001868.v1.p1).

Research involving human participants, their data, or biological material

Policy information about studies with human participants or human data. See also policy information about sex, gender (identity/presentation), and sexual orientation and race, ethnicity and racism.

Reporting on sex and gender	Sex was used as a covariate in the primary GWAS analysis. We relied on the genetic-sex inferred from genotype data. Nevus count difference across sex was assessed using ordinal logistic regression method. Then, sex-stratified GWAS meta-analysis was performed to explore the sex-differentiated effects of genetic variants. Findings are relevant to both men and women.
Reporting on race, ethnicity, or other socially relevant groupings	Our meta-analysis represents individuals of European ancestry.
Population characteristics	This meta-analysis is conducted using 14 cohorts of participants from Australia, Netherlands, UK and USA. Age and sex distributions were varied and full description of study cohorts are provided in Supplementary Methods and Supplementary Table 2. In brief, across the new cohorts,(QSkin Sun and Health Study (QSKIN) Phase I, QSKIN Phase II and Australian Genetics of Depression Study (AGDS) studies) the proportion of females ranged from 57% to 76%, and participant ages ranged from 18 to 93 years.
Recruitment	This study does not involve direct recruitment of study participants and used previously collected data. Please see main text and supplementary notes for the study recruitment details of all studies included in our analysis
Ethics oversight	The Human Research Ethics Committee of QIMR Berghofer Medical Research Institute gave ethical approval for this work as well as the ethical approval for the QSkin Sun and Health Study (QSkin I: P1309 and P2034 and QSkin II: P3434) and Australian Genetics of Depression Study (AGDS: P2118). The Kidskin Young Adult Myopia Study (KYAMS) was approved by the Human Research Ethics Committees of the University of Western Australia (RA/4/1/6807). All study participants over 18 years provided written informed consent; for those under 18 years consent was obtained from parents.

Note that full information on the approval of the study protocol must also be provided in the manuscript.

Field-specific reporting

Please select the one below that is the best fit for your research. If you are not sure, read the appropriate sections before making your selection.

☒ Life sciences ☐ Behavioural & social sciences ☐ Ecological, evolutionary & environmental sciences

For a reference copy of the document with all sections, see nature.com/documents/nr-reporting-summary-flat.pdf

Life sciences study design

All studies must disclose on these points even when the disclosure is negative.

Sample size	The number of datasets was determined by the availability of all eligible cohorts with phenotype and genotype data at the time the study was initiated. Eleven cohorts from a previous meta-analysis and three newly available cohorts were included forming the largest GWAS meta-analysis for nevus count to date. It is generally accepted that GWAS meta-analysis of this scale provide sufficient power to detect biologically relevant genetic susceptibility loci.
Data exclusions	Individuals were excluded based on; non-European ancestry, high genotyped missigness and having melanoma history. Genetic variants with minor allele frequency lower than 1% or imputation quality lower than 0.5 were excluded.
Replication	We conducted GWAS meta-analysis using all available independent GWASs. Meta-analysis results were not directly replicated, but the effect size estimated from meta-analysis were used in PRS (Polygenic Risk Score) to predict outcomes in independent cohorts. Additionally, our study was found previously reported nevus/melanoma/pigmentation-associated variants and genes alongside novel loci.
Randomization	Randomization is not applicable in GWAS analysis
Blinding	Blinding is not applicable in genome-wide association studies as analyses were conducted on pre-existing, de-identified GWAS datasets.

Reporting for specific materials, systems and methods

We require information from authors about some types of materials, experimental systems and methods used in many studies. Here, indicate whether each material, system or method listed is relevant to your study. If you are not sure if a list item applies to your research, read the appropriate section before selecting a response.

Materials & experimental systems

n/a	Involved in the study
☒	☐ Antibodies
☒	☐ Eukaryotic cell lines
☒	☐ Palaeontology and archaeology
☒	☐ Animals and other organisms
☒	☐ Clinical data
☒	☐ Dual use research of concern
☒	☐ Plants

Methods

n/a	Involved in the study
☒	☐ ChIP-seq
☒	☐ Flow cytometry
☒	☐ MRI-based neuroimaging

Plants

Seed stocks	N/A
Novel plant genotypes	N/A
Authentication	N/A
